# Establishing a living systematic review of characterisation and parameter reporting in lithium-ion and lithium–sulfur cathode research

**DOI:** 10.1007/s44373-026-00099-1

**Published:** 2026-04-10

**Authors:** Liam Bird, Yiheng Shao, Boyi Pang, James Robinson, Paul Shearing

**Affiliations:** 1 The ZERO Institute, Holywell House, Osney Mead, Oxford, OX2 0ES UK; 2 Department of Engineering Science, University of Oxford, Parks Road, Oxford, OX1 3PJ UK; 3 The Faraday Institution, Quad One, Becquerel Avenue, Harwell Science and Innovation Campus, Didcot, OX11 0RA UK; 4 Advanced Propulsion Lab (APL), Marshgate, London, E20 2AE UK

## Abstract

Advancing energy storage and decarbonising transportation while improving today’s Lithium-ion technology remain crucial, however it is equally important to pioneer new battery systems, such as lithium–sulfur (Li–S), which can meet future demands. Despite decades of progress in materials synthesis and characterisation, inconsistencies in parameter reporting and testing standards continue to limit reproducibility and cross-comparison across the battery literature. Here, we present a systematic meta-analysis of nickel–manganese–cobalt oxide (NMC) Li-ion and Li–S cathode systems, using a Python-based graphical user interface to standardise data extraction for electrode fabrication, electrochemical testing, and physical characterisation. The analysis reveals that even for the higher-technology readiness level (TRL) NMC chemistry, key experimental parameters such as electrode thickness, testing temperature, and formation protocols are frequently omitted. Both battery systems also exhibit inconsistencies in analytical procedures and ambiguity in the understanding of underlying principles when interpreting characterisation results. Our findings highlight systematic under-reporting of scalable manufacturing parameters and essential comparability metrics across both chemistries. This work establishes a cross-chemistry benchmark for transparent and reproducible reporting, providing a foundation for accelerating the translation of materials-level advances toward higher TRLs.

**Supplementary Information:**

The online version contains supplementary material available at 10.1007/s44373-026-00099-1.

## Introduction

Increasing global demand for electrified transport, including electric vehicles (EVs), and distributed electricity generation has prompted research interest in rechargeable batteries. This includes intercalation-based lithium-metal oxide chemistries (Li$_{x}$CoO$_{2}$, LCO, and Li$_{x}$Ni$_{y}$Mn$_{z}$Co$_{(1-y-z)}$O$_{2}$, NMC) which are established in consumer electronics and personal EVs. However, developments in lightweight batteries such as conversion chemistry-based lithium–sulfur (Li–S) are required to enable weight-critical applications including aerospace and road-freight transport [1].

Battery research articles typically focus on a specific component (i.e. anode, cathode, or electrolyte), however interactions within the cell system mean that the effect of each component cannot be assessed in isolation, even in a half-cell configuration where one electrode is replaced by lithium metal. Journals including ACS [2], Batteries Europe [3], Joule [4], the Journal of Power Sources [5], and Wiley [6] have published guidelines for key metrics to include in articles reporting developments in electrode formulations to ensure transparency in comparing electrochemical performance. These guidelines are voluntary, and previous meta-analyses of Li–S literature [7–9] found inconsistency in which of these metrics are reported. Additionally, a variety of physical, chemical, and electrochemical characterisation methods are used to measure the properties of electrode materials, however different journal articles report different combinations of characterisation techniques [10] applied to different measurands (for example, electrode materials vs. electrodes containing binder and additives).

Given its widespread commercial adoption with gigafactory-scale fabrication facilities [11], NMC has a high technology readiness level (TRL) assessed at 9 on NASA’s definition scale [12]. Meanwhile, Li–S is approaching commercial-scale manufacture, with prototypes and demonstrators available, but no mass-market adoption at time of writing, placing Li–S at a TRL of 5–6 [13]. Their distinct operating regimes make it likely that Li–S and NMC batteries will play complementary, rather than directly competitive, roles in electrification [1], and the unique technical challenges posed by each chemistry will necessitate different innovation trajectories to reach a similar TRL. Acknowledging these differences, this meta-analysis investigates whether the degree of standardisation in Li–S research is comparable to NMC, and seeks to identify whether lessons from the higher TRL NMC can be applied to advance Li–S development.

This meta-analysis investigates the frequency with which different recommended metrics, and which combinations of characterisation methods, are reported for battery chemistries with different TRLs. Data corresponding to the metrics recommended in journal guidelines (summarised in Table S1) from 200 articles were collated. With a view to consistent comparison, this analysis focuses on published work primarily focused on the electrochemically active material in the cathode (i.e. positive electrode) for both Li–S and Li-ion cells, referred to as CAM. In Li–S cells, this includes the conductive material used to form a composite with the sulfur (typically carbon), and in Li-ion cells this refers to the NMC. Section 2 presents a short overview of the current status of each battery chemistry, highlighting turning points in the development of each. Section 3 covers the methodology for collating the data from the 100 Li–S and 100 NMC-focussed articles, and defines key categories and nomenclature used in the later analysis. The collated data is analysed with a focus on electrode synthesis (Sect. 4), electrochemical testing (Sect. 5), and physical characterisation (Sect. 6). Throughout these analysis sections, the macro-scale trends identified are supported by $\sim$3 representative examples drawn from the articles included in the meta-analysis: these example lists are not exhaustive, but intended to indicate the order of magnitude of parameter values, typical combinations of components, or frequently-sought characterisation outcomes. The complete dataset including further examples is available in the Supplementary Information. Finally, Sect. 7 presents a discussion and outlook of the extent to which currently-available data is useful in reproducible battery research, and identifies opportunities for improvement in future publications.

## Status of Li-ion and Li–S technology

NMC is currently the dominant Li-ion battery chemistry for light-duty EVs, including cars, accounting for $\sim$ 60% of EV battery sales in 2022 [14] and projected to continue to dominate the market alongside lithium iron phosphate (LFP) to 2050 [11, 14]. The combination of Ni, Mn and Co results in improved thermal stability compared to Li$_{x}$CoO$_{2}$, reduced degradation compared to the effects of cation mixing in Li$_{x}$NiO$_{2}$, and improved gravimetric capacity compared to Li$_{0.5}$Mn$_{2}$O$_{4}$ [15–18]. Following initial developments in the use of layered transition metal oxides and spinels in Li-ion cathodes from 1980 onwards [19–22], and specifically NMC 111 from 1999 onwards [23, 24], research focus and commercialisation have shifted towards nickel-rich chemistries [11] including NMC 532 [25], 622 [26], 811 [27, 28], and 955 (i.e. LiNi$_{9}$Mn$_{0.5}$Co$_{0.5}$ [29]. The motivations for decreasing Co content include human rights and environmental concerns [30] and geopolitical sensitivity associated with Co mining, and the aim of developing Ni-rich chemistries is therefore to ensure supply chain security and minimise cost. Additional current research focuses include mitigating capacity degradation, including the use of single-crystal NMC [31] to mitigate cracking, and by stabilising the interface between the active material and electrolyte by using coatings [16] or core-shell structures with a Ni-rich core and increasing Mn concentration towards the surface [17]. Other approaches seen in this meta-analysis include low concentrations of dopants including Mg (e.g. [32–34]), Al (e.g. [2, 35, 36]) and Mo (e.g. [2, 37]), with the aim of stabilising the layered transition metal oxide structure during (de-)intercalation of Li$^{+}$.

Meanwhile, Li–S batteries are currently moving from laboratory scale demonstrators [13] to pilot-line scale production (e.g. [38–41]), predominantly targetting applications in small-scale air transport. By contrast to the intercalation mechanism in conventional Li-ion electrodes, Li–S cells use the reversible chemical conversion of solid sulfur (S$_{8}$) to solid lithium sulfide (Li$_{2}$S) *via* a series of soluble lithium polysulfides (LiPS). Although this gives Li–S cells a higher theoretical gravimetric capacity of 1675 mAh g$^{-1}$, compared to $\sim$277 mAh g$^{-1}$ for NMC, the diffusion of the soluble LiPS into electrical isolation from the cathodic matrix results in capacity degradation, a key challenge to their widespread adoption. To address this issue, research initially focussed on tuning electrolyte solubility to mitigate diffusion [42, 43], however renewed interest in Li–S research was catalysed by Ref [44]’s demonstration of an optimised conductive cathodic matrix, consisting of a templated hierarchically porous carbon. Optimising the morphology of the cathodic matrix to prevent LiPS diffusion remains a key focus of Li–S research [10], alongside incorporation of functional groups and dopants [45–47]. Ongoing challenges to the widespread adoption of Li–S batteries include incompatibility of the widely-used electrolyte additive LiNO$_{3}$ with UN Transport Recommendations [48], and the offsetting of the high theoretical gravimetric capacity due to excess electrolyte utilisation [10, 49]. Approaches to this include going foregoing the solid–liquid-solid phase transformation by tuning the interaction between the host matrix and the electrolyte [50], however this meta analysis reflects the existing focus on host matrix morphology and ether-based electrolytes.

## Methodology

Data corresponding to recommended parameters collated from 5 voluntary reporting checklists, summarised in Table S1, were collected using a graphical user interface (GUI) implemented in a Python Jupyter notebook (available in Supplementary Information) to facilitate fast, standardised data entry for a large number of parameters, including pre-filled entries for the most common values. The GUI also includes a checkbox grid for characterisation techniques and corresponding measurands, grouped by data type and measurement probe, specifically: morphological (mainly qualitative, optical or electron imaging), structural (mainly X-ray derived), vibrational spectroscopy, pore size and surface area, and electrode composition. The measurands were classified as: the uncycled electrode (including the active material and, where present, the conductive carbon and binder coated on a metal current collector), *in-situ* or *operando* characterisation undertaken during electrochemical testing, and *post-mortem* characterisation of electrodes recovered from cycled cells. Additional measurands are the ‘raw materials’, which corresponds to the NMC powder in NMC-related articles, and generally refers to the conductive host matrix in the absence of sulfur in Li–S articles, while ‘composite’ is used specifically for the S/C composite in Li–S articles to differentiate this from the conductive host.

Parameter information was collated from a total of 200 articles (100 Li–S and 100 NMC articles), with data drawn from both the main text and supplementary information. Articles were identified using the Web of Science database and through citations in relevant review articles, with the most recent search performed on 17/08/2025. To be eligible for inclusion, articles were required to report the galvanostatic cycling capacity corresponding to a cathode under investigation, where the target of the investigation may be the method of synthesising the active material (e.g. S/C composite, NMC) or electrode manufacturing method (e.g. modifying quantity of conductive additive). For database searches, the following search combinations were used. Terms related to ‘electrolyte’, ‘separator’, ‘anode’, and life-cycle assessment and resource recovery were excluded from article titles to return articles whose central focus is on cathode materials development.

**Li–S**: “lithium–sulfur" OR“Li–S" OR “lithium-sulphur" OR “lithium sulfur" or“lithium sulphur" OR “Li S" (Topic) and cathode (All Fields) not electrolyte OR separator OR interlayer OR coating OR anode (Title) not “all-solid-state" OR “solid state" OR “solid electrolyte" (Title) not Review (Document Type)

**NMC**: nickel AND manganese AND cobalt AND battery (All Fields) or NMC OR NCM AND battery (All Fields) not NMC OR NCM (Author) not algorithm OR regression OR “life cycle analysis" OR “life cycle assessment" OR recovery OR sodium OR potassium (All Fields) not recycling OR “solid state" OR “battery management system" OR electrolyte (Title) not Review (Document Type)

## Electrode preparation

### Electrode slurry and coating

The electrode composition, including the ratio of CAM, binder, and conductive carbon additive (where used) is fundamental to reproducibility of reported results and to direct comparison of electrochemical results, especially for novel CAM. The content of these electrode constituents was reported by 96 %  of articles included in this meta analysis (the data is omitted in [51–53], and values are included in a previous referenced publication by one [36]), and is typically reported as relative contents of each constituent, e.g. 80: 10: 10 by weight CAM: binder: conductive additive. Assuming that the electrode slurry is homogeneous, in principle these ratios are relevant to all scales of manufacture. However, the electrode homogeneity is also affected by factors including the electrode mixing, coating, and drying processes, which may in turn be determined by the scale of electrode synthesis. For example, $\sim$10 s-100 s mg quantities of novel materials prepared in a laboratory are likely to be prepared using manual mixing or small planetary mixers, while optimising the mixing procedure using pilot-line scale equipment was shown to strongly affect the electrochemical behaviour of electrodes produced in $\sim$100 s mL batches [13, 54]. Consequently, the ACS [2]  and JPS [5]  voluntary checklists include the scale of electrode synthesis, however as shown in 1a, this is reported by 64  and 21  Li–S and NMC related articles respectively. The absolute quantities of electrode precursors are most commonly reported (45  Li–S articles, 10  NMC articles). For example, Ref [33] specifies the use of 2.079 g nickel nitrate and 5.072 g manganese nitrate (*inter alia*) when synthesising NMC, and Ref [55] reports the use of 1.2 g V$_{2}$O$_{5}$ and 2.5 g oxalic acid as precursors to forming hollow spheres which were used as a host matrix for Li–S cathodes. In the latter, although the absolute quantity of sulfur is not specified (the relative quantity, 80 wt%, is measured empirically), these precursor values are indicative of the total volume of electrode slurry and associated mixing conditions.

Where electrodes are coated on metal current collectors (rather than freestanding), the electrode structure is also affected by the electrode slurry viscosity during coating and mixing [56]. However, the solid content of the electrode slurry is reported by only 8 %  articles, including Refs [54] and [57] where the electrode slurry solvent was the subject of optimisation. Furthermore, Ref [57] demonstrates the significant effect of binder processing history on the electrochemical behaviour of Li–S electrodes, while Ref [58] investigates the effect of slurry solvent on the dispersion of electrode powder and consequent electrode structure. However few articles explicitly report the method of binder preparation, including the pre-dissolution of binder in solvent before mixing with the other dry components [20, 34, 57–59]. Refs [60, 61] specify the use of pre-dissolved binders (LA133 and polyvinylidene fluoride, PVDF), with Ref [61] specifying its viscosity. In other articles, the amount of solvent used is unspecified, in some cases referred to as “an appropriate amount" [62], or “enough" [63], indicating experimental experience in producing a slurry with suitable viscosity. The energy demand for electrode drying contributes approximately 12–13 % to the embodied energy of cell manufacture [54, 64, 65], and the replacement of NMP with non-toxic alternatives compatible with binders free from per- and polyfluoro alkyl substances (PFAS) is a research priority for both sustainable initial manufacturing and electrode recycling [66, 67]. Therefore, while the goal of adjusting the electrode slurry viscosity is to produce a homogenous electrode with a uniform thickness and/ or areal loading (discussed below) after removal of the solvent, the slurry solvent content and associated electrode drying conditions are useful parameters for reproducibility at the lab scale and are relevant for scale-up.

### Electrode thickness and calendering

**Fig. 1:**
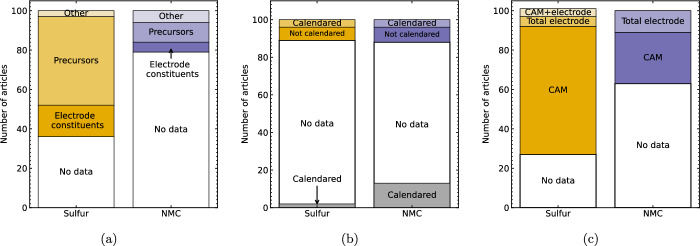
Frequency of reporting electrode synthesis parameters for Li–S and NMC articles. **a** Production scale, e.g. $\sim$mg - $\sim$g scale: Electrode constituents refers to materials used directly in cell, e.g. sulfur, NMC powder, and precursors refers to e.g. glucose used for carbonisation, NMC hydroxides. **b** Electrode thickness and calendering, where grey bars at bottom show reports of calendering without reporting the thickness. **c** Areal loading of cathode active material (CAM), total electrode including binder and additives, or reporting both CAM and total electrode values

The electrode thickness, porosity, and associated areal loading of CAM influence the cell-level gravimetric (Wh kg$^{-1}$) and volumetric (Wh L$^{-1}$) energy density. The ratio of both the mass and volume of CAM to auxiliary cell components, including the metallic current collector and the polymer separator, can be increased both by using thicker electrodes to achieve a higher areal loading of CAM (kg m$^{-2}$) and by increasing their volume density by calendering (compression with a defined load and temperature). In principle, the electrode thickness is a readily accessible measurand, however practical difficulties in measuring it are reflected by its reporting in 11  and 12  of Li–S and NMC articles respectively (see Fig. 1b). As previously noted in Ref [7], the areal loading of active material is reported more frequently (73 % of Li–S, 37 % of NMC articles), potentially because of its accessibility *via* electrode mass. Where the electrochemical performance is reported for multiple different areal loadings of electrodes with the same composition (e.g. NMC [68], Li–S [55, 57, 58, 69–76]), the effect of electrode thickness can be qualitatively inferred based on the correlation between greater thickness and higher areal mass loading.

In some instances, the thickness was indicated *via* the coating setting, typically Doctor blade height (Li–S [77], NMC [22, 61, 78–81]), although providing an indicative value, the realised thickness after solvent evaporation depends on factors including the solid content of the slurry (discussed above). In other instances, the electrode thickness was specified based on the known dimensions of a template or precursor, for example those used for freestanding electrodes for Li–S cells [82, 83], or measured directly using a micrometer or SEM (Li–S [84–86], NMC [59, 87]). However, increasing the thickness of Li–S cathodes may limit their rate capability and reversible capacity utilisation due to longer diffusion distances for Li$^{+}$ ions perpendicular to the cathode/ separator interface [88]. Furthermore, additional flocculent carbon black per unit area introduces more surface area requiring contact by the electrolyte: Ref [60] calculated that, due to the higher carbon black content of Li–S vs. Li-ion electrodes, their porosity may be as high as 70% compared to c. 30% [89] for Li-ion cathodes. Percolating this pore network requires additional electrolyte, potentially offsetting beneficial weight savings from reduced areal requirement for auxiliary components. On one hand, porosity and concomitant electrolyte requirement, in addition to the electronic conductivity (due to contact between electrode particles), can be controlled via calendering. For example, Ref [60] demonstrated a linear increase in conductivity when decreasing the electrode porosity to 50% in Li–S electrodes. On the other hand, decreased porosity is accompanied by increased tortuosity of connections between pores where tortuosity is defined as the ratio of the total path taken length though the porous material to the linear distance between the path’s start and end points [90]. Precipitation of Li$_{2}$S during discharge may therefore block these pathways, decreasing the reversible capacity compared to lower tortuosity electrodes [90], which is supported by Ref [60]’s observation of lower wettability and lower pore visibility in calendered vs. uncalendered electrodes. 1 article included in this meta-analysis reported the electrode tortuosity quantitatively, calculated from EIS measurements of symmetrical (cathode/ cathode) coin cells [58]. The tortuosity value may also be determined from tomographic data, for example using tools such as TauFactor [91], however as shown in Sect. 6 this data is frequently unavailable to researchers, likely due to high cost and the time-consuming nature of these techniques, with X-ray computed tomography reported by 5 Li–S [57, 58, 76, 92, 93] and 1 NMC article [59], and focussed ion beam scanning electron microscopy (FIB-SEM) reported by 2 Li–S [57, 94] and 7 NMC articles [32, 95–100].

Despite the control offered by calendering over the electrode thickness, as shown by the grey bars in in Fig. 1b, 15  articles report the use of calendering without reporting the thickness. Instead, the calendering parameters are reported in terms of pressure (e.g. 3 kN [2], 10 N mm$^{-1}$ [101]): this is consistent with the Joule voluntary reporting checklist [4], the only one to specify calendering parameters. Although one outcome of calendering is improving the uniformity of electrode thickness following coating, controlling the interaction with the electrolyte *via* porosity and tortuosity directly affects the electrochemical behaviour of the cell, therefore the results of calendering may be reported as a function of electrode porosity [60, 102]. For scale-up, further parameters including the speed and heat applied are required for reproducibility.

## Electrochemistry

### Statement of nominal capacity

**Fig. 2:**
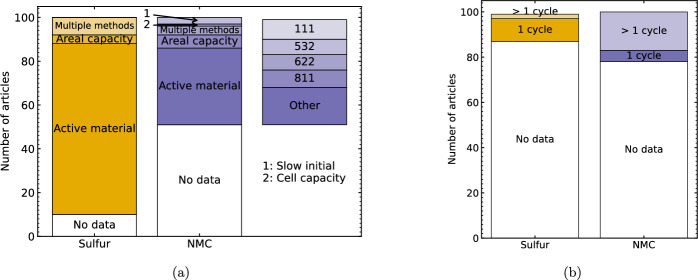
Frequency of reporting parameters for gravimetric cycling. **a** Basis for calculating cell capacity or C rate, where right bar shows number of articles where assumed capacity is reported for each NMC stoichiometry. ‘Active material’: theoretical capacity in mAh g$^{-1}$, ‘Areal capacity’: mA cm$^{-2}$. **b** Use of slow formation cycle prior to long-term cycling

Reporting galvanostatic cycling results for the electrode material under test was a pre-requisite for inclusion of an article in this meta-analysis. The galvanostatic rate used was specified in terms of current (mA or mA g$^{-1}$), or C rate (where 1C = 1 charge/ discharge in 1 h). Despite this, and the recommendation by four of the publisher’s voluntary reporting guidelines ([2, 4–6]), as shown in Fig. 1b, the gravimetric capacity used to determine the C rate or current density was specified by 49  NMC articles. By contrast, 90  Li–S articles explicitly state the theoretical capacity of sulfur, likely because its high gravimetric capacity compared to LCO is a key motivator for its development (with one article referring to sulfur’s theoretical capacity as “marvellous" [73]). The less frequent reporting in NMC articles may be due to the undefined practical maximum (de)-intercalation of Li in NMC (i.e. the stoichiometry of x in Li$_{x}$Ni$_{y}$Mn$_{z}$Co$_{1-y-z}$O$_{2}$) for novel NMC stoichiometries, while the requirement for 16 Li$^{+}$ ions for the chemical reduction of S$_{8}$ to Li$_{2}$S is well-defined. To this end, 3  NMC article reported the use of a slow initial cycle (Sect. 5.3) to determine the practical capacity of the cell for use in setting the C rate. However, as shown in Fig. 2a, the theoretical capacity is specified with approximately equal frequency for different types of NMC, including ‘other’ stoichiometries than the commonly used types specified. While the theoretical capacity of Li$_{x}$Ni$_{y}$Mn$_{z}$Co$_{1-y-z}$O$_{2}$ can be easily calculated assuming that *x*=1, where there is no explicit basis for C rate calculation and the current density is unspecified, there is no self-contained means to directly compare the galvanostatic current densities between articles. However, when converting reported current densities to C rates in Sect. 5.2 using an assumed theoretical capacity of 277 mAh g$^{-1}$ for NMC, integer- or near-integer values of N for for C/N or NC were obtained, suggesting that this value is used for calculation even where not explicitly stated.

### C rate ranges

**Fig. 3:**
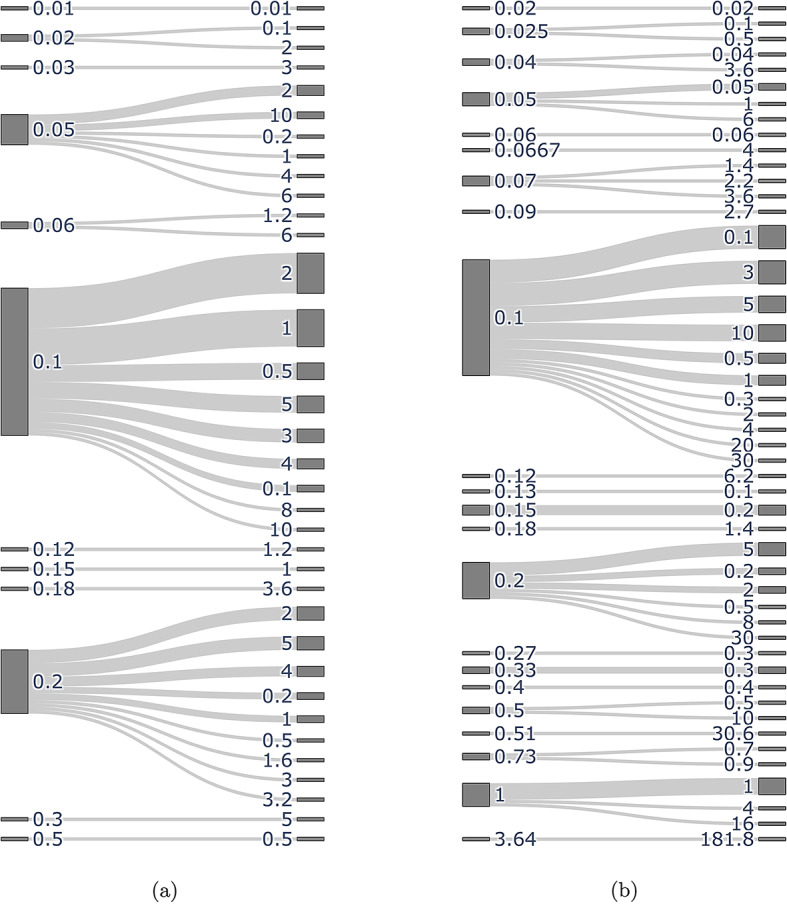
Minimum and maximum C rates reported for **a** Li–S and **b** NMC articles. Left columns show minimum reported rate, right columns show maximum reported rate (for either continuous or progressive rate capability cycling). Joining line thickness proportional to number of articles reporting each combination of minimum/ maximum rates

The minimum (left columns) and maximum (right columns) C rates are summarised in Fig. 3, with 0.1C and 0.2C the most commonly used minimum rates used in the analysed articles. Where the galvanostatic cycling conditions were specified in terms of current density (mAh g$^{-1}$), these values were converted to C rate using assumed theoretical capacities of 1675 mAh g$^{-1}$ and 277 mAh g$^{-1}$ for Li–S and NMC articles respectively. When comparing the plots, note that 1C corresponds to $\sim$275 mA g$^{-1}$ for NMC (see discussion above), and 1675 mA g$^{-1}$ for Li–S. The C rates summarised in Fig. 3 represent a mixture of long-term cycling at a continuous rate, and step-by-step rate capability tests to reach higher values such as 10C. A breakdown of the frequency of use of different rate capability protocols for Li–S cells is available in Ref [7], however the focus of this work is comparing whether the most materials-level characterisation conditions are representative of targets for commercial cells. The US Advanced Battery Consortium (USABC) has published targets of 350 Wh/kg at cell level using C/3 (i.e. 20 min charge) for EVs in general [103], and Refs [104, 105] propose targets of 3–6 C for electric vertical take-off and landing (eVTOL) vehicles with pulse power requirement up to 12 C. However, other roadmaps (e.g. [1, 106, 107]) specify targets for power density in terms of W/kg or W/L, referring the mass or volume to the cell or pack-level, making direct comparison to C rate reporting challenging.

**Fig. 4:**
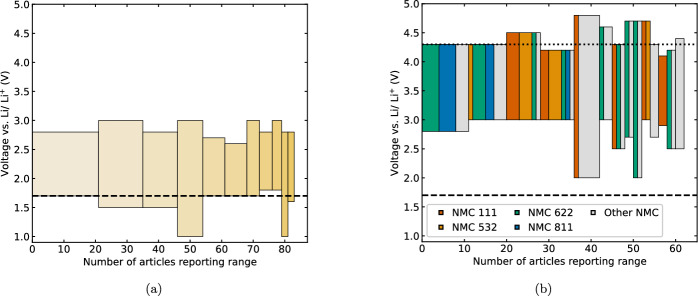
Minimum and maximum voltage ranges for galvanostatic cycling reported by at least one article for **a** Li–S articles and **b** NMC articles. Dashed line in **a** at the minimum voltage where LiNO$_{3}$ is stable, dashed and dotted lines in **b** show window where capacity degradation is minimised in NMC full cells

Furthermore, unlike the C rate, the energy and power density are functions of cell voltage. Figure 4 shows the frequency of using different upper and lower cut-off ranges used in more than 1 article for Li–S and NMC, with the vertical height spanning the voltage range specified and the width corresponding to the number of reports. The operating windows are plotted on the same y-axis to highlight the likelihood of Li–S and NMC fulfilling complementary, rather than competing, roles in applications. In Fig. 4a, a lower cut-off of 1.7 V is indicated with a horizontal line, corresponding to the voltage at which the commonly-used (73 % of Li–S articles [7]) additive LiNO$_{3}$ undergoes irreversible decomposition [108, 109]. Similarly, Fig. 4b indicates the upper cut-off voltage at 4.3 V, corresponding to oxygen evolution from the NMC structure which contributes to an irreversible capacity loss [110]. Notably, 85 % of the NMC-related articles included in this meta-analysis are half-cells with lithium anodes, so were unaffected by processes at the graphite anode outside of this voltage window in commercial cells [111]. The bars in Fig. 4b are further broken down by NMC stoichiometry, with ‘other’ including approximately equal numbers of cells with x$\ge$ 0.8 and x<0.8 for LiNi$_{x}$Mn$_{y}$Co$_{(1-x-y)}$O$_{2}$. When operating in the same conditions with upper cut-off voltage $\le$ 4.15V, cells with high nickel content cathodes (x$\ge$0.8) have higher gravimetric energy than those with lower nickel content (NMC622, NMC532, NMC111) [16], while cells with the same gravimetric capacity operated over wider voltage ranges have higher energy density. However, when charging cells with high nickel content to higher upper cut-off voltages, initial gains in charge capacity are offset by accelerated degradation [81, 112, 113], associated with processes including a phase transition at 4.1 V vs. Li/ Li$^{+}$, resulting in volume change and loss of structural integrity [113–115]. This is consistent with the data for NMC811 in Fig. 4b, where all reported high-nickel samples were charged to a maximum of 4.3V. The use of upper cut-off voltages above 4.3V for ‘other’ NMC compositions likely reflects the data collection process, where the maximum and minimum voltages within each article are recorded, and therefore captures articles where different voltage windows are used for non-standard NMC compositions (including high nickel) to demonstrate stability or degradation (e.g. [81, 116, 117]).

### Formation cycles

The C rates shown in Fig. 3 represent the rates used for long-term cycling stability evaluation, however, Fig. 2b shows that 22  NMC articles and 7  Li–S articles additionally used slow initial cycles. This typically involved cycling at a median rate of 0.10  or 0.05  for NMC and Li–S articles respectively, with the highest number of cycles specifically used for ‘formation’ being 10 for NMC [116]. Where used in Li–S articles, the slow initial cycles aim to ‘activate’ the sulfur. While the commonly-used melt-infiltration method of S/C composite synthesis aims to form a thin layer of sulfur at the electrochemically active interface, as evidenced by XRD, crystalline $\alpha$-S$_{8}$ commonly exist in these as-produced porous structures [7, 44]. Here, the sulfur in the ‘core’ of these ionically and electronically insulating particles initially isolated from the interface. The slow formation cycle therefore aims to enable the reduction of sulfur and subsequent oxidation of LiPS to sulfur while minimising the influence of diffusion and/ or rate limitations that may occur at higher current densities [118]. In commercial Li-ion cell manufacture, including NMC cells, an initial slow formation cycle is used to produce a stable solid-electrolyte interphase (SEI) and cathode-electrolyte interphase (CEI), which mitigate ongoing decomposition reactions between the electrolyte and electrically conductive interfaces of the electrodes [119]. The infrequency of using a formation cycle in the materials development-focussed articles included here may be attributed to the majority use of half-cells (85% in this dataset) with lithium metal anodes, rather than commercially representative full cells with lithiated graphite anodes (12% in this dataset), leading to different interphase requirements which may lead researchers to forego equivalent formation cycling to that used in full cells. However, at higher TRL, confirming the effect of the formation cycle on the engineered cathode is relevant in the context of optimising formation conditions: in commercial NMC manufacture, the formation and ageing process accounts for $\sim$20–25% of the total energy demand for cell manufacture [65, 119, 120], so optimising these steps is instrumental in reducing the embodied energy of cells.

### Temperature

**Fig. 5:**
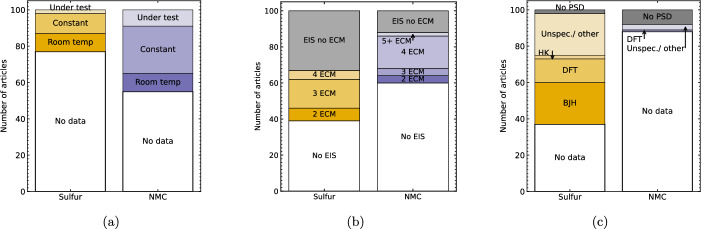
Frequency of reporting electrochemical parameters. **a** Temperature during cycling (‘under test’: capacity investigated as a function of temperature), **b** Electrochemical impedance spectroscopy (EIS) and number of components in series in equivalent circuit model (ECM), **c** N$_{2}$ adsorption results for BET (all coloured bars) and method of pore size distribution determination (HK: Horwath Kazoe, DFT: Density functional theory, BJH: Barret-Joyner-Halenda, ‘other’ includes mercury intrusion porosimetry)

All of the reporting guidelines surveyed for this meta-analysis recommend reporting the environmental temperature used during cycling, however as shown in Fig. 5a this is reported by 23% of Li–S and 45% of NMC articles respectively. The reported temperatures were classified as ‘room temperature’ where this is explicitly stated but no temperature value or means of direct monitoring and control is reported, ‘constant’ where a temperature value is specified and is assumed to be directly monitored and/ or maintained, and ‘under test’ where the electrochemical performance of the cells is investigated as a function of cycling temperature. The specified ‘constant’ temperature is typically close to room temperature (e.g. 25± 5$^{\circ }$C). However, Ref [121] demonstrated the difference between cycling Li-ion pouch cells in temperature controlled chambers at 25$^{\circ }$C versus ambient conditions, where self-heating in the absence of active air circulation caused the cells in ambient conditions to operate at 27$^{\circ }$C. Although this is a small variation, on a similar scale to diurnal temperature fluctuations in a laboratory, in Li-ion full cells this affected cell longevity due to the competing effects on lithium plating and SEI formation around a pivotal temperature of 25$^{\circ }$C in commonly-used electrolytes in Li-ion cells. While temperature ranges of $\sim$25–60 $^{\circ }$Cwere reported in the NMC-focussed articles in this meta-analysis [37, 122–124] the Li–S examples included here used a wider range from $\le$ - 10–50 $^{\circ }$C[92, 125], with the low temperature testing likely motivated by the target applications of Li–S cells in aerospace environments [126]. Li–S cells show a similar trend to Li-ion, with higher temperatures corresponding to similar or higher initial capacity compared to 25 $^{\circ }$C  but more rapid capacity degradation, however this trend is caused by different physical processes in Li–S cells. The lower capacity retention at high temperatures is associated with increased overpotential, particularly affecting the lower discharge voltage plateau [92, 127], while for Li–S cells with similar electrodes and electrolytes the upper discharge plateau capacity and overpotential are less affected at lower temperatures than the lower plateau, corresponding to the slower kinetics [44] of the conversion of Li$_{2}$S$_{6, 4}$ to Li$_{2}$S$_{2}$ and Li$_{2}$S compared to the initial reduction of S$_{8}$ to long-chain LiPS [128]. The role of kinetics in the temperature dependence of Li–S performance is also demonstrated by Ref [125], who demonstrated a reduced capacity loss at lower temperatures by optimising the conductive host matrix to mitigate the higher impedance at the electrolyte/ electrode interface encountered at lower temperatures. Therefore, future reporting would benefit from inclusion of the temperature of the lab during electrochemical cycling, including the range of ambient temperatures where this is not directly controlled, to enable valid comparison between studies.

### Electrolyte

**Fig. 6:**
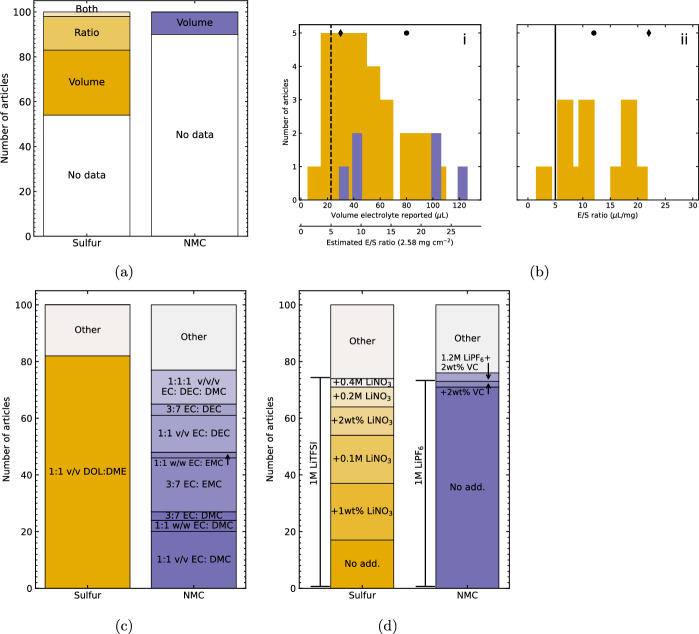
Frequency of reporting electrolyte-related parameters. **a** Electrolyte content by volume ($\mu$L) or ratio ($\mu$L mg$^{-2}$ active material). **b** Volume of electrolyte (i) and E/S ratio (ii). Offset *x* axis in (i) shows estimated E/S ratio for average sulfur areal loading and 15 mm diameter electrode; markers show two reports where both volume and ratio are reported. Vertical line indicates 5$\mu$ L E/S ratio target from Ref [49]. **c** Electrolyte solvent combinations. DOL: 1, 3-dioxolane, DME: 1, 2 dimethoxyethane, EC: ethylene carbonate, DEC: diethyl carbonate, DMC: dimethyl carbonate, EMC: ethyl methyl carbonate. v/v: volume ratio, w/w: weight ratio. **d** Salt and electrolyte additive combinations. LiTFSI: Lithium bis(trifluoromethanesulfonyl)imide, VC: vinyl carbonate, No add.: No additive.‘Other’ includes all electrolyte solvent combinations with 1 report

Figure 6a shows the frequency with which the electrolyte content of prototype cells is reported in articles included in this meta-analysis. The high theoretical gravimetric capacity of Li–S cells, based on the mass of the active material S$_{8}$, can be offset by the relatively large contribution to the cell-level mass from the liquid electrolyte, leading to the identification of target electrolyte/ sulfur (E/S) ratios to ensure that Li–S cells have competitive gravimetric energy density at the cell level. These targets are typically specified in $\mu$L electrolyte per mg S$_{8}$, including 5 $\mu$L mg$^{-1}$ [49], 3 $\mu$L mg$^{-1}$ [13], or 2 $\mu$L mg$^{-1}$ in a pouch cell [10]. The use of excess electrolyte in Li–S cells minimises the influence of capacity degradation processes associated with higher electrolyte viscosity, preventing percolation of the microporous structure, where the concentration of dissolved S$_{8}$ and LiPS is higher [129, 130]: therefore, the dominant processes limiting capacity attainment and retention are attributable to features of the cathode matrix under development. This enables evaluation of the pore size distribution, surface area, and other parameters without the electrolyte limiting reversible sulfur utilisation. By contrast, the electrolyte accounts for a smaller proportion of the mass of practical NMC-based cells ($\sim$10 % [131], 1.3 g Ah$^{-1}$ [132] approximately equivalent to 4.7:1 electrolyte: NMC by mass) due to the higher mass density of the NMC cathode and graphite anode than their counterparts in Li–S cells. The electrolyte content was most commonly reported as the absolute volume per coin cell, rather than as an E/S ratio, which is attributable to the practical accessibility of this value, however the offset x-axis of Fig. 6b shows an estimated corresponding E/S ratio based on the mean sulfur areal loading (2.58 mg cm$^{-2}$) and coin cell electrode diameter of 1.5 cm. The skew towards lower E/S ratios in Fig. 6b, combined with the increased frequency of E/S ratio and/ or electrolyte volume reporting in Li–S cells after c. 2019 (Fig. 10a) suggests that authors are more likely to report instances of proactively aiming to minimise the E/S ratio, while omitting the quantity used in cells with excess electrolyte.

Figure 6 compares the frequency with which different electrolyte solvents (a) and salt/ electrolyte additive combinations (b) are used in the articles included in this meta-analysis. As shown in Fig. 6c, 81 Li–S articles use 1:1 v/v 1,2-dimethoxyethane: 1,3-dioxolane (referred to as DOL:DME) as the electrolyte solvent, while Li-ion cells with NMC cathodes use a more diverse range of solvent combinations. DOL: DME is an established electrolyte solvent for Li–S batteries with conventional liquid electrolytes due to the solubility for LiPS provided by DME [133], and the role of DOL in forming an ionically conducting, electronically insulating layer on the lithium metal anode to prevent ongoing electrolyte decomposition [43, 109, 134]. In Li-ion cells with NMC cathodes, the cyclic molecule ethylene carbonate (EC) is commonly used in conjunction with linear carbonates including dimethyl carbonate (DMC), diethyl carbonate (DEC), or ethyl methyl carbonate (EMC). EC has a high dielectric constant and is consequently conducive to LiPF_6_ dissociation, and forms an effective SEI on graphite anodes [135, 136]. However, EC has a melting point of 36.4 $^{\circ }$C, so is solid at room temperature conditions [135, 136]. To form a liquid electrolyte with sufficiently low viscosity to wet the porous electrodes, linear carbonates are used as co-solvents, including in commercial electrolytes such as LP30 (1:1 v/v EC: DMC with 1 M LiPF_6_) [137] and LP57 (3:7 EC/EMC with 1 M LiPF_6_) [138], corresponding to the most frequently used solvent combinations shown in Fig. 6c.

By contrast, as shown in Fig. 6d the use of electrolyte salt and additives is more consistent in the Li-ion samples reviewed in this meta-analysis, with 75 articles using 1 M LiPF_6_ salt without additives, while Li–S samples consistently use 1 M LiTFSI electrolyte salt with a greater variety of LiNO_3_ concentrations. LiNO_3_ is used to promote the growth of a continuous passivating film on the lithium anode to prevent ongoing reactions between the anode and dissolved polysulfides in the electrolyte, thereby mitigating the LiPS shuttle effect [134, 139]. Additionally, LiNO_3_ catalyses the oxidation of long-chain LiPS to S_8_, lowering the required driving force for the reaction and thereby protecting the integrity of pathways in the electrically conducting host matrix [140]. However, due to gas evolution by LiNO_3_ at $\le$40 $^{\circ }$C [141], LiNO_3_ is not compatible with UN Recommendations on the Transport of Dangerous Goods [48], meaning that effective elimination of LiNO_3_ from Li–S electrolyte is necessary for widespread adoption of Li–S batteries.

### Cyclic voltammetry

**Fig. 7:**
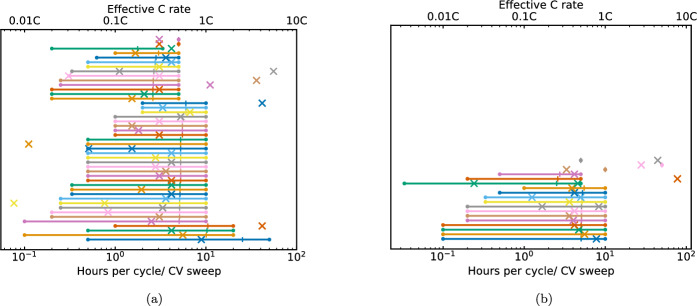
Comparison of range of C rates (spans, with linear midpoint indicated by vertical marker) and equivalent duration of CV sweep in hours (crosses) reported in the same article: one span per article where both CV and galvanostatic cycling are reported for **a** Li–S and **b** NMC electrodes

Although the application of a constant current in galvanostatic cyclic is more representative of discharging a battery through a load, complementary information can be obtained from cyclic voltammetry (CV). Galvanostatic cycling and CV are therefore inequivalent physical processes, however comparing the total duration of CV sweeps with galvanostatic C rate indicates whether conclusions about reaction kinetics are derived from tests which are representative of target galvanostatic operating conditions. Figure 7 compares the duration of CV sweeps (crosses) with C rates (ranges terminated by dots) in articles included in this meta-analysis. In 32% and 11% of Li–S and NMC articles respectively, a single CV rate was reported which fell within the same range of durations as the galvanostatic cycling rates. Figure 7 also shows that CV is more widely used in Li–S research than NMC. By contrast, dQ/dV analysis is widely employed in NMC research but was only found in 1 Li–S article [142]. In the Li-ion intercalation system, dQ/dV analysis highlights phase transitions and degradation processes associated with the sloping voltage profile [143]. However in the Li–S system, the two-plateau discharge curve can be intuitively interpreted for information about the overpotential and relative capacity derived from each step of the reaction.

The use of multiple CV sweep rates can also be used to infer the Li$^{+}$ diffusion coefficient, and the range of sweep rates over which the electrochemical behaviour is mass transport rather than rate limited, where a higher diffusion coefficient over a wider operating window is indicative of cells with higher cycling rate capability. Among the articles included in this meta-analysis, the Randles-Ševčik equation relating CV sweep rate (*v*) to diffusion coefficient was reported by 1 Li–S [144] and 1 NMC [145] article. While both confirmed that the peak current value ($i_{p}$) increased linearly with $v^{{1}/{2}}$, 1 condition on the validity of the Randles-Ševčik equation and recommended in the JPS voluntary reporting guidelines [5], in both cases the peak overpotential increased with increasing $v^{{1}/{2}}$ thereby limiting the equation’s strict applicability. Further examples of multiple CV sweep rate use were to confirm the reversibility of Li–S conversion reactions [146], and to differentiate between Faradaic and non-Faradaic electron transfer processes in NMC [147]. Alternative methods of inferring the diffusion coefficient of novel electrode materials seen in this meta-analysis included galvanostatic intermittent titration (GITT), all in NMC articles [98, 148, 149], and electrochemical impedance spectroscopy (EIS, see Sect. 5.7), used for this purpose in 3 NMC articles [62, 150, 151].

### Electrochemical impedance spectroscopy

EIS can be used to infer the number of electron transfer processes in an electrochemical system, grouped by their resistive and reactive response at different perturbation frequencies. As a non-destructive process, it can be applied to cells undergoing conventional galvanostatic cycling to investigate changes in the presence and relative contribution of different interfaces to electron transfer processes, subject to cells being allowed to equilibrate before measurement. Equivalent circuit models (ECMs) can provide an intuitive representation of electron transfer processes, although their interpretation is ambiguous as multiple ECM configurations can be used to fit the same data. The non-unique attribution of ECMs to EIS spectra is reflected by the variation in the number of ECM elements reported by articles included in this meta-analysis, shown in Fig. 5b. Here, the number of ‘elements’ refers to the number of ECM components in series. Here, an ‘element’ may therefore refer to a resistor modelling the Ohmic resistance of the electrolyte, a Warburg element, or Voigt circuit with a resistor and constant phase element in parallel with each other: despite the differences between these ‘elements’, this is used here as a bases for comparison because each series ‘element’ typically represents a modelled interface or electron transfer process. In NMC articles, 18  use 4 element ECMs, typically comprising an Ohmic resistor, two Voigt elements, and a Warburg element to represent electrolyte resistance, SEI and charge transfer impedance, and bulk diffusion respectively (e.g. [123, 147, 152]). Li–S articles use ECMs with an Ohmic resistor, typically with 2 Voigt elements to represent the passivation layer at the lithium anode and charge-transfer resistance between the electrolyte and LiPS (e.g. [117, 153, 154]), although the representation of bulk diffusion varies, with some articles using Voigt or constant phase elements (CPE) (e.g. [155]) and others using Warburg elements (e.g. [60, 117]). In semi-quantitative usage, ECMs provide comparisons of the relative contributions of interfacial processes to cell impedance, for example by tracking the change of impedance contributions with similar characteristic frequencies (and therefore likely similar physical interpretation) throughout a time series of EIS spectra for the same cell [155]. Similarly, 2 articles in this meta-analysis illustrated the change in physical phenomena contributing to cell impedance before and after cycling using ECMs fitted to EIS spectra acquired before and after cell cycling [70, 125], incorporating an additional Voigt element to represent charge transfer impedance after an initial formation cycle (see Sect. 5.3).

The variation in ECM fitting between articles is partly attributable to the aforementioned ambiguity in interpretation: where distinct physical processes contributing to the impedance have similar characteristic frequency, they may be indistinguishable from each other in the Argand plane. Although not applied in any of the articles included in this meta-analysis, since c. 2017 in Li-ion [156] and c. 2022 in Li–S, distribution of relaxation times analysis has been increasingly applied to differentiate features in the time domain which overlap in the frequency domain while reducing reliance on the analyst’s judgement about the number of features to fit. For example, Ref [156] identified distinct contributions from the current collector and SEI from a broad, overlapping peak in Li-ion anodes, while Ref [157] identified up to 8 distinct contributions to Li–S impedance, including up to 4 from the overlapping broad semi-circle at low-mid frequencies. While ECM fitting tools are frequently embedded in potentiostat software, open-source DRT analysis tools such as the DRTtools MATLAB add-on [158] are available. In addition to ambiguity of interpretation, a further reason variation between the ECM models selected by different articles is indicated by Ref [159], where EIS spectra for multiple cells from the same batch are compared, showing qualitatively different spectra throughout their cycling life, including different relative contributions from impedance contributions attributed to different physical processes. However, consistent attribution of impedance contributions elucidated by DRT showed that the contribution from SEI was dominant in predicting capacity fade and cell failure in Li–S cells with lithium metal anodes. Analysis of the trends in impedance contributions, particularly those strongly correlated with cell degradation, may therefore yield more transferable information than direct comparison of EIS spectra or discrete fitting values due to the variability between cells with nominally identical parameters. However, the variability in models fitted to derive these parameters highlights the challenges with direct comparison of quantitative impedance values reported in different articles.

## Physical characterisation

### Measurand distribution

**Fig. 8:**
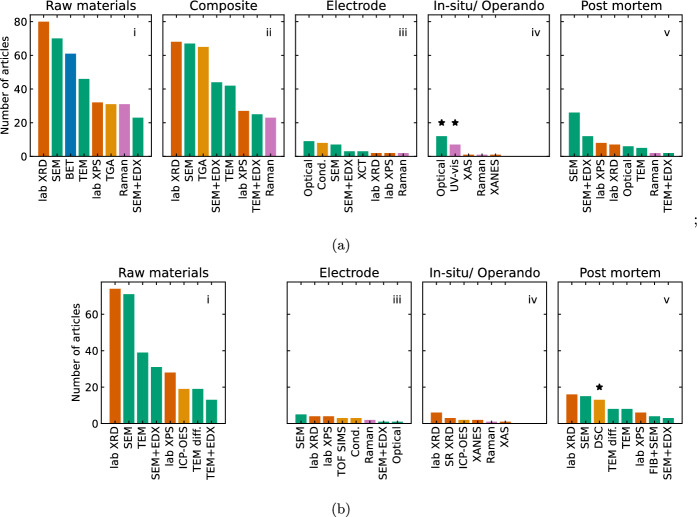
Summary of most frequently applied techniques to different measurands for Li–S (**a**) and NMC (**b**) related articles, showing the 8 most commonly used for each measurand. Raw materials: for Li–S, refers to host matrix separate from active sulfur; for NMC, refers to NMC powder. Composite: for Li–S, refers to sulfur/ host composite (not applicable to NMC, bii intentionally omitted). Electrode: uncycled electrode including binder, additive, and current collector. *In-situ/ operando*: cell with electrolyte undergoing electrochemical testing, or electrolyte and electrode undergoing chemical reaction (see text). *Post-mortem*: electrode removed from cell after electrochemical cycling, characterised without electrolyte or anode

In Fig. 8, all of the measurands are shown on the same y-axis to highlight the prevalence of characterising the raw materials and occasional reporting of *post-mortem* electrodes. This is attributable to the challenges with requiring specialised cells for *in-situ*, with geometry different from standard coin cells, and experimental compatibility with time-resolved measurement [160, 161]: synchrotron X-ray diffraction (XRD), X-ray absorption spectroscopy (XAS), and X-ray absorption near edge spectroscopy (XANES) are among the techniques reported for *in-situ* characterisation in this meta-analysis (Fig. 8a iv and 8b iii). For Li–S, optical photographs and UV–vis in Fig. 8b iv and marked with an asterisk were categorised as *‘in-situ’*, but correspond to photographs comparing the colour change of LiPS solutions [86, 117, 142, 146, 162–167], or UV–vis spectra of LiPS solutions [73, 164, 166, 168–170] following adsorption of LiPS species onto host matrices to demonstrate their efficacy in preventing inventory loss by diffusion. Similarly, differential scanning calorimetry (DSC) is marked with an asterisk and shown as *post-mortem* as it is typically applied after lithiation of the NMC and deconstruction of the cell. By contrast, uncycled electrodes appear to be more accessible measurands than *in-situ* cells undergoing electrochemistry, however the focus of articles in this meta-analysis was CAM development, and therefore direct comparison of synthesis outputs is facilitated by characterising them in the absence of binder and low mass density, high volume fraction carbon black additives. Notably, where electrical conductivity is reported (13 % of Li–S articles, 9 % of NMC articles), it is most commonly reported for the uncycled electrode: this is rational because it includes the electrically conductive network incorporating conductive carbon additives. As previously noted [7], consistent reporting of the electrical conductivity of uncycled, as-synthesised electrodes does enable direct comparison between reports, but does not necessarily represent changes relevant to operating electrodes, such as binder swelling due to electrolyte interactions [171].

### Evaluating active material synthesis

**Fig. 9:**
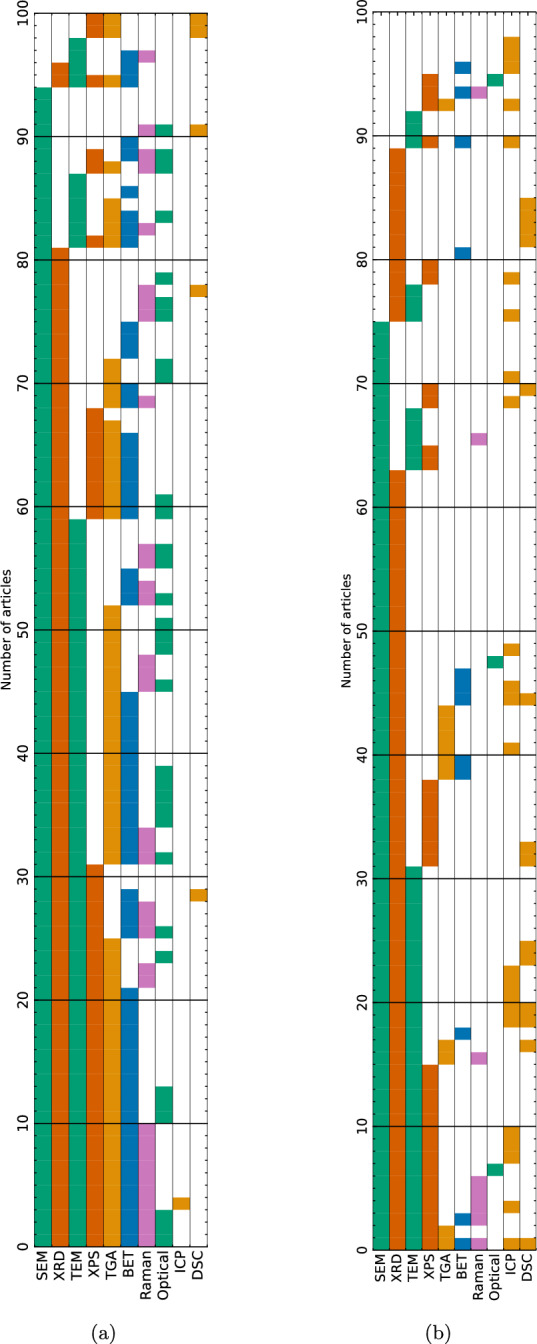
Most frequently used combinations of characterisation techniques for **a** Li–S and **b** NMC related articles with a focus on the 10 most commonly reported techniques. Each row corresponds to one article, with colour blocks in corresponding columns indicating techniques used in each article. Combinations are grouped with the most frequently used at the bottom. SEM: Scanning electron microscopy, XRD: X-ray diffraction, TEM: transmission electron microscopy, XPS: X-ray photoemission spectroscopy, TGA: Thermogravimetric analysis, BET: N$_{2}$ absorption surface area analysis, ICP: inductively coupled plasma, DSC: differential scanning calorimetry. SEM and TEM include imaging and energy dispersive X-ray (EDX) spectroscopy

Figure 9 shows that the most frequently used characterisation techniques for both Li–S and NMC are SEM and XRD, providing complementary information about the $\sim$1 $\mu$m scale morphology and the $\sim$Å  scale crystal structure. However, the different information requirements are shown by the application of techniques to different measurands in Fig. 8. The need to confirm the efficacy of S/C composite synthesis technique in producing an homogenous layer of sulfur at the host/ electrolyte interface is seen by comparing Fig. 8ai to Fig. 8bii: SEM with EDX (generally EDX mapping), is more frequently applied to the S/C composite than the raw materials to demonstrate the uniformity of sulfur distribution, while 66  of 80  Li–S articles reporting XRD for the raw materials also report it for the S/C composite. As previously discussed [7], this XRD comparison is frequently used to demonstrate whether the composite synthesis method results in bulk, crystalline $\alpha$-S$_{8}$ or a thin, amorphous layer [44]. Similarly, as shown in 8ai and ii, Raman spectroscopy is used to compare the S/C composite with the raw materials in 22  articles: unlike the attenuation of XRD peaks associated with amorphous S$_{8}$ relative to $\alpha$-S$_{8}$, the Raman spectrum depends only on the D$_{4d}$ symmetry of S$_{8}$ molecules. When investigating carbon-based conductive host matrices, Raman spectroscopy is typically used to infer the effect of processing, including heating for the melt-infiltration of sulfur (e.g. [172–174]), *via* the I(D)/I(G) ratio, although decreasing I(D)/I(G) ratio is not uniquely correlated with increasing graphitic domain size [175].

Furthermore, 65  of Li–S articles report TGA for the S/C composite, with 30  reporting TGA for both the composite and ‘raw materials’. Where it is reported for ‘raw materials’ in Fig. 8a, this is often to compare the mass loss profile of the S/C composite to sulfur to validate the temperature of mass loss, and/ or to compare the slightly delayed period of sulfur loss after incorporation into the S/C composite as evidence of encapsulation of the sulfur in the matrix (e.g. [44, 93, 153]). TGA is utilised to provide confirmation of S/C composite composition following melt infiltration, *in-situ* chemical formation by reduction of Na$_{2}$S$_{2}$O$_{3}$ or Na$_{2}$S$_{x}$, or solution of sulfur in CS$_{2}$ or toluene: although the concentration or quantities of input reactants can be known, losses during synthesis are otherwise difficult to quantify. Since the metal oxide constituents of NMC electrodes are more refractory, inductively coupled plasma (ICP) is used more commonly in NMC-focussed articles to confirm the composition of raw materials, however a minority (9) NMC articles do report TGA with differential thermal analysis for example, to quantify the oxygen content [176] or to demonstrate mass loss corresponding to phase transitions [25] or calcination processes [177, 178]. Furthermore, given the small quantities typically used in these materials development-focussed articles (see Sect. 4.1), $\sim$0.1 mg losses contribute significantly to measurement uncertainty, making analytical confirmation useful.

SEM and XRD are also the characterisation techniques most frequently applied to NMC, however by contrast to the binary presence/ absence indication of $\alpha$-S$_{8}$ peaks in S/C composite XRD spectra, Rietveld refinement is routinely used in NMC-focussed articles to characterise its structural geometry, reflecting the need for reversible (de-)intercalation of Li$^{+}$ ions rather than the non-topotactic chemical conversion of sulfur. This is supported by the more frequent use of TEM with diffraction in NMC-related articles, and the use of *in-situ* XRD to monitor the structural distortion of the inter-layer *c*-spacing in layered oxides compared to the intra-layer *a*-spacing. During the first charge, the *c*-spacing initially increases due to repulsion between O$^{2-}$ sites in the absence of Li$^{+}$, before decreasing towards the end of charge due to attraction between O$^{2-}$ and transition metal ions (e.g. [27, 179–181]). Subsequent XRD spectra acquired for NMC electrodes, both in modified cell configurations for *in-situ* characterisation and extracted for *post-mortem* characterisation from conventional cells (Fig. 8biv, v) show that these structural changes remain stable after the first cycle (e.g. [27, 180, 182]).

### Structural characterisation

XPS is reported by 46 % of Li–S and 32 % of NMC articles in this meta-analysis, although distinct information is sought for each battery chemistry and each measurand. In Li–S, examples of XPS use include probing the extent of host matrix/ active material interaction and the role of oxygen containing functional groups (e.g. [183, 184]), with Ref [57] using the surface sensitivity of XPS to demonstrate the role of binder distribution on host matrix/ active material interaction. Unlike other characterisation techniques including XRD and EDX, XPS differentiates between lithium-containing species including Li$_{2}$S$_{x}$ (x$\ge$1) formed during cycling, and Li$_{2}$S$_{x}$O$_{y}$-containing compounds indicative of irreversible reaction products between the sulfur and electrolyte. In *post-mortem* samples extracted from cycled Li–S cells, XPS has therefore been used to identify deposits responsible for pore-blocking (e.g. [60]) and to verify the efficacy of functional groups and dopants in anchoring LiPS (e.g. [185, 186]). An analogous application of XPS in *post-mortem* NMC electrodes (and their corresponding anodes) is the characterisation of the CEI and SEI, where analysis of differentiated carbon- and oxygen-containing group peaks provides insights into the contribution of decomposed carbonate electrolyte solvents and LiPF$_{6}$ salt. This has been used to infer the role of electrochemical cycling conditions (e.g. [187]) and the influence of cathode interface morphology and composition (e.g. [99]) on SEI/ CEI composition, which in turn affects the interfacial impedance and electrolyte degradation processes in the cell. In addition to interfacial interactions, in NMC electrodes, XPS is applied to identify the valence states of Ni, Mn, and Co (e.g. [51, 188]) and in some instances to quantify the proportion of surface species in each valence state, for example the relative content of Ni$^{2+}$ vs. Ni$^{3+}$ [189, 190].

### Pore size distribution and surface area

The different energy storage mechanisms in Li–S and NMC require different priorities in quantitative physical characterisation techniques, including pore size distribution of the carbon host matrix in Li–S, and (less frequently) particle size distribution in NMC. The specific surface area (SSA) and pore size distribution are typically determined using N$_{2}$ adsorption isotherms, with the SSA determined using the multipoint BET (Brunauer-Emmett-Teller) equation, although 2 Li–S [74, 94] and 1 NMC [191] articles report mercury intrusion porosimetry. In Li–S, the SSA and particle size distribution are most commonly measured for the carbon host matrix (Fig. 8a), with 20  articles contrasting this with measurements for the composite to demonstrate the degree of pore filling by the sulfur (e.g. [44, 153, 192]). However, since the volatility of sulfur means that the degassing process of heating under vacuum to remove adsorbed water cannot be used prior to measurement, values for composites are likely to represent underestimates [44]. In addition to the initial sulfur particle size [118, 193], the pore size distribution and SSA of Li–S electrodes is correlated with the amount of electrolyte required to wet the surface [49] (see Sect. 5), whereas the particle size distribution of NMC dictates the diffusion distance of Li$^{+}$ ions to intercalant at the centre of the article, and possible cracking-based degradation mechanisms [194]. Consequently, 6 NMC articles report laser-diffraction or dynamic light scattering particle size determination methods [79, 87, 97, 176, 195, 196], (plus 2 Li–S articles [94, 168]). Meanwhile, 5 NMC articles [2, 36, 79, 152, 197] and 6 Li–S articles [71, 84, 166, 198–200] report particle size distributions determined SEM micrographs, e.g. statistics from 50 measured particles. In Li–S, the most commonly used method of calculating pore size distribution is the BJH (Barrett-Joyner-Halenda) equation (23  articles), which may underestimate the pore size for pores less than 10 nm [201], however two articles [77, 125] specified both the BJH and HK (Horvath and Kawazoe) which enables the determination of micropores $\le$2 nm [201, 202]. While density functional theory (DFT)-based methods are compatible with micropore determination [201], adoption and specification of consistent a pore size determination method such as the most widely-reported BJH would enable correlation of electrochemical behaviour with quantifiable trends in SSA and pore size distribution, despite systematic limitations in the micropore range.

## Discussion

**Fig. 10:**
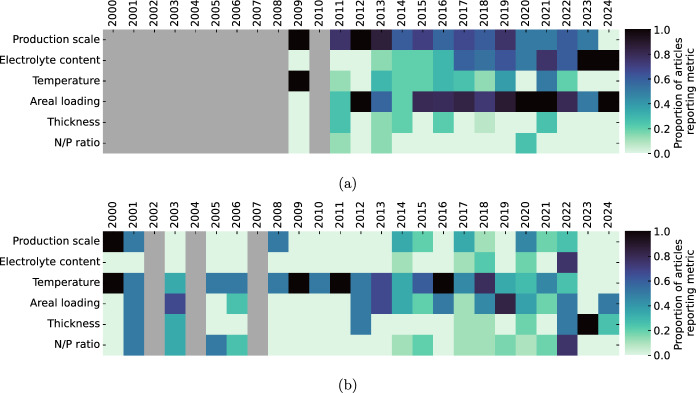
Proportion of articles for each year included in the meta-analysis reporting the listed metrics. (Grey indicates no articles available in that year within the meta-analysis). Some years contain data from single articles, and the figure represents only data from within the dataset of 200 articles

In quantifying the frequency with which different synthesis and testing parameters are reported for NMC and Li–S, despite the higher TRL of NMC electrodes, it is clear that there is still space for standardisation in the suite of reported parameters for both chemistries. Given the motivation of direct comparison between articles, this is likely to be a self-fulfilling trend. The frequency of reporting selected parameters with time is represented in heat maps in Fig. 10, showing an overall trend of increased frequency and completeness of parameter reporting with time. The 100 articles for each battery chemistry currently included in this meta-analysis represent a small fraction of the total available, hence the heat maps in Fig. 10 are indicative of a sample rather than quantifying an overall trend, however the data collated for this work is intended as a foundation for a larger database. The parameters shown in these heat maps were selected for their ease of measurement, as a subset of recommended parameters from the voluntary reporting guidelines (recommended parameters that are currently universally reported, for example wt% mass of binder, cell format, and electrolyte composition are omitted from these time series). In principle, the production scale and electrolyte volume do not require any additional measurement, but their reporting would lead to more reproducible research. In a benchmarking study, Ref [203] compared solid-state cells made with identical constituents but using different assembly protocols in different labs, including different orders of assembly and different assembly environments, with the obtained results showing wide variation, demonstrating how the standardisation of routine protocols which are not directly associated with the materials development affect the reproducibility of the conclusions drawn. Similarly, Ref [204] demonstrated the effect of “trivial” parameters in cell assembly on Li–S cells specifically, including the pressure used during cell crimping and auxiliary component thickness, with marked differences in stable capacity and early cell failure. Many of the factors investigated by Ref [204] are included in journal voluntary reporting checklists, specifically in checklist [5], however of the 200 articles included in this meta analysis only 1 [197] included a battery-specific voluntary reporting checklist in its supplementary information, with a further article [100] including a reproducibility checklist that is non-specific to battery research. Despite the availability of the voluntary reporting checklists, correspondence with 3 of the 5 journals indicates that their implementation is not currently routinely tracked. Notably, the single example where a checklist is included does not report every suggested metric: even more frequent reporting of incomplete checklists would identify common blind spots, and may prompt the standardisation of commonly-used parameters even where they remain non-optimised.

Naturally, in these articles selected for their focus on cathode materials development, non-optimised parameters associated with auxiliary cell components which would not be directly compatible with scale-up to commercial cells, may go unreported because they appear to detract from the competitiveness of the material [205]. Approximately 20 stoichiometries of NMC-related materials other than established/ integer compositions were included in this meta-analysis, ranging from high Ni (e.g. LiNi$_{0.96}$Mn$_{0.01}$Co$_{0.03}$O$_{2}$ [206]) to high Mn (e.g. LiNi$_{0.13}$Mn$_{0.54}$Co$_{0.13}$O$_{2}$ [190]). Meanwhile, Li–S host matrix morphologies ranged from ‘starfish-like’ [207] to ‘French fries-like’ [163], and the materials explored ranged from bio-derived soybean hulls [174] and waste tea [208] to highly ordered covalent organic frameworks [209]. It is unrealistic to require all reports of novel materials to demonstrate their feasibility directly in lean electrolyte, low N/P, prototype cells following scalable synthesis procedures when such studies typically work with half-cells using $\sim$100 mg of the candidate material (see Sect. 4). However, using electrolyte/ active ratio as an example, it is likely that excess electrolyte is being used in all such exploratory cells, and it may be possible to identify materials-specific advancements among novel materials tested in conservative, but comparable conditions where the volume of electrolyte is consistently reported.

Beyond the frequently reported parameters and established characterisation techniques discussed in Sects. 5 and 6, there are opportunities for extracting additional useful information from routine measurements. More frequent reporting of the parameters included in Fig. 10 would both aid reproducibility and enable investigation of correlations between these parameters and cell performance. A further example related to Li–S galvanostatic cycling data is the ratio between the capacity attained at each discharge plateau, and the respective polarisation of each plateau, which is often described qualitatively. However, 6 articles quantified these values and identified trends throughout the cycling life of cells, enabling identification of materials with higher sulfur utilisation and lower polarisation [58, 74, 93, 144, 163, 163].

The database collated during this work catalogues values and references for key parameters, and is available in the Supplementary Information: there is scope for expansion beyond the extant 200 articles. In addition to cross-referencing within the database to identify trends, the database can also serve as a directory for identifying input parameters for numerical modelling. For example, where no single article contains all of the required parameters, filtering the database by cell chemistry, electrode composition, and cell configuration (i.e. half cell/ full cell) can be used to find mutually compatible sets of parameters.

## Conclusions

This meta-analysis collated data on the electrode synthesis, electrochemical testing, and physical characterisation reported for 100 Li–S and 100 NMC-focussed research articles published between 2000 and 2024 to identify trends in data reporting with the progression of TRL. Among the parameters with relevance to both battery chemistries, there is variability in whether the values are reported for each electrode type, and where they are present, there is further variability in the methods of applications and data analysis protocols.

Comparing the reporting frequencies for the technologies reveals opportunities for more complete reporting without additional experimental work in future articles. Although the ultimate aim of materials-level research is the scale-up and commercialisation of improved battery technology, this leads to the omission of fundamental parameters from research publications as they do not appear optimised for commercial viability. However, reporting such values, even where non-optimised, would enable direct comparison of electrochemical results from different studies, and improve reproducibility as each inter-dependent cell component is optimised. Li–S articles are more likely than NMC to report the areal loading of active material (73% for Li–S vs. 37% for NMC), which can be determined using the average mass of electrodes of known geometric area. This is indicative of the electrode thickness, which is required for reproducibility, but is infrequently measured directly (11% Li–S and 12% NMC). Similarly, Li–S articles are more likely to report the scale of synthesis (64% for Li–S vs. 21% for NMC), which is also likely to impact reproducibility *via* its effects on slurry mixing homogeneity and and the availability of suitable equipment to enable scaled-up synthesis within the required parameter tolerances. For other parameters, simple additional measurements and records could contribute to enhanced reproducibility, including the temperature of cells during cycling, which is presently reported more frequently in NMC (45%) than Li–S (23%). Although controlled, constant temperature cycling provides the most directly comparable results, the sensitivity of cell performance to temperature means that reporting the minimum, maximum, and average temperature experienced by cells in ‘room temperature’ laboratories is instructive when comparing results between articles.

Despite the focus on cathode development in this meta-analysis, the complex interaction between cell components means that the reporting of electrochemical results from cells with novel cathode chemistry in combination with non-standard electrolytes and non-standard voltage ranges makes it difficult to identify correlations between individual parameters and cell performance, as demonstrated for Li–S cells in Ref [7]. Although more widespread use of reporting checklists such as Refs [2–6] will aid improvements in reproducibility, this meta-analysis does not identify ‘beach head’ parameters to prioritise for reporting in future articles as key determinants of reproducibility. Of the 200 articles surveyed, only 1 reported electrochemical data averaged from multiple cells [101], otherwise it is assumed that each article reports data from a single cell for each parameter combination. However, known from the experience of experimentalists in the battery development field, and documented quantitively in Refs [159, 203], cells produced using materials from the same batch and assembled using nominally similar procedures may have different initial capacities, rates of capacity decay, and failure mechanisms. Therefore, more frequent reporting of the electrochemical data from repeat samples in future articles would provide an immediate insight into the sensitivity of cell performance to auxiliary parameters and aid identification of priority parameters for standardisation.

The data collation and analysis framework established in a previous report [7] and expanded here provides a flexible basis for comparing other trends in the battery literature. Having evaluated materials-level trends and variability in Li–S coin cells, there is scope for expanding this methodology to pilot-line and laboratory-scale cell development.

## Supplementary Information

Below is the link to the electronic supplementary material.

Supplementary file 1 (pdf 146 KB)

Supplementary file 2 (csv 282 KB)

## Data Availability

All data aggregated and analysed for this publication are available as a.csv file in the Supplementary Information. The code developed for the graphical user interface is available at the following URL: https://github.com/LiamBird/meta-characterisation.git.
