# Supplementary material for: Establishing a living systematic review of characterisation and parameter reporting in lithium-ion and lithium–sulfur cathode research
Source: Discov Electrochem. 2026 Apr 10;3(1):35. doi: 10.1007/s44373-026-00099-1 (PMC13104760; doi:10.1007/s44373-026-00099-1)
Supplement: Supplementary file 1 — (pdf 146 KB) [file 44373_2026_99_MOESM1_ESM.pdf]

Supplementary information

Table S1: Collated voluntary reporting checklist for similar metrics recommended by >1 journal and/ or referred to directly in main text. The checklist entries shown here are summarised from specific requirements listed individually on each list, grouped together as overarching categories where appropriate. Note that the Batteries Europe document is not presented in explicit checklist format, and efforts have been made to summarise the recommendations for comparison here.

Topic	JPS [1]	Joule [2]	ACS [3]	Wiley [4]	Batteries Europe [5]
**Positive/ working electrode**					
Scale of synthesis	✓		✓		
Supplier and purity of reagent	✓		✓		✓
Reaction conditions for synthesis	✓				
Mass composition of electrode	✓	✓	✓	✓	
Areal mass loading of electrode	✓	✓	✓		
Calendering pressure		✓			
**Cell components**					
Cell type and configuration	✓	✓	✓	✓	✓
Number of separators used	✓			✓	
Electrode thickness/ component thickness	✓	✓		✓	
Electrolyte volume vs. active material	✓	✓			
N/P ratio or active material excess	✓	✓	✓		✓
Mass of all components		✓			
Cell type (half/ full)		✓	✓	✓	
Current collector	✓			✓	
**Electrochemical testing**					
Environmental temperature	✓	✓	✓	✓	✓
Pressure applied to cell			✓		✓
Voltage range used for galvanostatic	✓		✓		✓
Nominal capacity	✓	✓	✓		✓
Capacity vs. full electrode/ full cell	✓	✓	✓		
Pre-activation	✓	✓	✓		
For CV: linearity of i_p vs $v^1 2$	✓				
Capacity for at least 3 cells		✓			

Table S2: Parameters collated from each article using graphical user interface, grouped by parameter category and data type. Default pre-filled values are shown underlined.

Parameter	Numerical	Free text	Boolean	Options
**Cell format**				
Anode materials		Other		Lithium metal, graphite, silicon
Cathode materials		Other		Sulfur, NMC811, NMC622, NMC532, NMC111, Other NMC, LFP/NMC blend, LCO
Cell type		Other		Coin, Swagelok, Pouch
Electrolyte volume (μL)	✓			
Electrolyte ratio	✓			
N/P electrode balance	✓		Reported?	
**Electrode processing**				
Production scale		Indicate scale	Reported?	
Commercial active material			Y/N	
Electrode composition reported				Binder, Additive, Active material, electrolyte solvent: solid ratio
Electrode thickness	✓			Coating setting, calendaring setting, direct measurement (e.g. micrometer), SEM, inferred from mass, precursor/ template thickness, other
Calendaring			Y/N	
Total electrode loading		Value with units		
Active material loading (mg cm^{-2})	✓			
Percentage active material in slurry	✓			
**Galvanostatic**				
Temperature				Not reported, Constant (non room temp), Room temp, under test
Theoretical capacity basis				Active material, areal/mass, cell, slow/ initial formation
Activation/ formation cycle	Rate, No. cycles	Other		
Voltage cutoffs	Min/ max		Multiple ranges	
C Rates	Min/Max			
Current density	Min/Max			mA g^{-2}, mA cm^{-2}
CC/CV step				CV during charge, CV during discharge
Multiple cells with statistics				Yes (in article), Yes (in SI), No
**Cyclic voltammetry (CV)**				
CV reported?			Y/N	
Voltage range	Min/ Max			
Sweep/ scan range	Min/ Max			
Diffusion by Randles-Sevcik and demonstration of linear relationship				Reported and checked, Reported not checked, Not reported
**Electrochemical impedance spectroscopy**				
EIS reported			Y/N	
Equivalent circuit model				None, 2 component, 3 component, 4 component, 5+ component

Table S3: Characterisation techniques recorded using checkboxes for each article for each measurand. micr. = microscopy, diffr. = diffraction, spectr. = spectroscopy
Measurands: Raw materials, composite (Li-S only), Electrode, In-situ/ operando, Post-mortem

**Morphology**	**Structure**	**Vibration**	**Electrode properties**	**Area/ porosity**
• SEM: Scanning electron micr.<br>• SEM+EDX: Scanning electron microscopy+Energy dispersive X-ray spectr.<br>• TEM: Transmission electron micr.<br>• TEM+EDX: Transmission electron micr. +Energy dispersive X-ray spectr.<br>• Transmission electron micr. + diffrac.<br>• Optical<br>• XCT: X-ray computed tomography<br>• Focussed ion beam+Scanning electron micr.	• lab XRD: X-ray diffrac.<br>• lab XPS: X-ray photoelectron spectr.<br>• lab XRF: X-ray fluorescence<br>• NMR: Nuclear magnetic resonance<br>• Neutron (imaging/ diffrac.)<br>• XAS: X-ray absorption spectr.<br>• EXAFS: Extended X-ray absorption fine structure<br>• SR XRD: synchrotron X-ray diffr.<br>• SR XRD: synchrotron radiography<br>• SR XRF: synchrotron X-ray fluroescence<br>• XANES: X-ray absorption near edge spectr.	• Raman spectr.<br>• FTIR: Fourier transform infra-red spectr.<br>• UV-vis spectr.	• 4-point conductivity<br>• Other conductivity<br>• TGA: Thermogravimetric analysis<br>• DSC: Differential scanning calorimetry<br>• ICP: Inductively coupled plasma<br>• TOF SIMS: Time of flight secondary ion mass spectr.	• BET/ MBET (Brunauer Emmett Teller)<br>• Density functional theory<br>• BJH (Barrett Joyner Halenda)<br>• HK (Horvath Kawazoe)<br>• Unspecified pore size distribution

Table S4: Sources for rarely-specified parameters. Due to the different measurement techniques and different measurands, measured values are omitted to avoid inconsistent comparison between articles. References corresponding to the main text reference list are included as a directory for difficult to find information

Parameter	Value	Ref
Formation period at OCV before cycling	$\leq$ 6 hours	[6–9]
	6-12 hours	[10–14]
	12-24 hours	[15, 16]
	> 24 hours	[17]
Conductivity	**Sulfur**	
	4-point probe	[18–21]
	DC polarisation	[22]
	Resistance quoted	[23]
	Impedance	[24]
	Other/ unspecified	[25–27]
	**NMC**	
	4-point probe	[28–30]
	DC polarisation	[31]
	Impedance	[32]
	Other/ unspecified	[33, 34]
Electrode thickness	**Sulfur**	
	Doctor blade coating setting	[35]
	Calendering	[22, 24, 36]
	Precursor/ template	[20, 37]
	Other, inc SEM	[6, 38, 39]
	**NMC**	
	Doctor blade coating setting	[12, 15, 40–44]
	Calendering	[45]
	Other inc. SEM	[7, 46, 47]

References

[1] J. Li, C. Arbizzani, S. Kjelstrup, J. Xiao, Y.-y. Xia, Y. Yu, Y. Yang, I. Belharouak, T. Zawodzinski, S.-T. Myung, R. Raccichini, and S. Passerini, "Good practice guide for papers on batteries for the Journal of Power Sources", Journal of Power Sources **452**, 227824 (2020).

[2] A. K. Stephan, "Standardized Battery Reporting Guidelines", Joule **5**, 1–2 (2021).

[3] Y.-K. Sun, "An Experimental Checklist for Reporting Battery Performances", ACS Energy Lett. **6**, 2187–2189 (2021).

[4] *Wiley VCH battery reporting checklist.*

[5] M. Margherita, A. Aurora, and E. Quartaone, *Batteries Europe Guidelines on common reporting methodology*, Batteries Europe, 2025.

[6] Z. Tao, Z. Yang, Y. Guo, Y. Zeng, and J. Xiao, "Plane Double-Layer Structure of AC@S Cathode Improves Electrochemical Performance for Lithium-Sulfur Battery", Front. Chem. **6** (2018).

[7] R. J. Tancin, B. Özdoğru, N. S. Dutta, D. P. Finegan, and B. J. Tremolet De Villers, "Direct reuse of graphite and lithium nickel manganese cobalt oxide (NMC) recovered from ultrafast-laser ablation debris in Li-ion battery electrodes", Journal of Power Sources **596**, 234027 (2024).

[8] S. Neudeck, F. Walther, T. Bergfeldt, C. Suchomski, M. Rohnke, P. Hartmann, J. Janek, and T. Brezesinski, "Molecular Surface Modification of NCM622 Cathode Material Using Organophosphates for Improved Li-Ion Battery Full-Cells", ACS Appl. Mater. Interfaces **10**, 20487–20498 (2018).

[9] C. Dillard, S.-H. Chung, A. Singh, A. Manthiram, and V. Kalra, "Binder-free, freestanding cathodes fabricated with an ultra-rapid diffusion of sulfur into carbon nanofiber mat for lithium sulfur batteries", Materials Today Energy **9**, 336–344 (2018).

[10] S. Yari, L. Bird, S. Rahimisheikh, A. C. Reis, M. Mohammad, J. Hadermann, J. Robinson, P. R. Shearing, and M. Safari, "Probing Charge Transport and Microstructural Attributes in Solvent- versus Water-Based Electrodes with a Spotlight on Li–S Battery Cathode", Adv. Energy Mater. **14**, 2402163 (2024).

[11]X. Zhang, S. Yang, Y. Chen, S. Li, S. Tang, D. Shen, W. Dong, and D. Hao, “Effect of phosphorous-doped graphitic carbon nitride on electrochemical properties of lithium-sulfur battery”, Ionics **26**, 5491–5501 (2020).

[12]H.-Y. Wang, S.-L. Mei, X.-L. Tan, B.-H. Lu, N. Li, and Z.-B. Wang, “Unveiling the particle-feature influence of lithium nickel manganese cobalt oxide on the high-rate performances of practical lithium-ion batteries”, Journal of Alloys and Compounds **1010**, 177774 (2025).

[13]J. W. Kim, J. J. Travis, E. Hu, K.-W. Nam, S. C. Kim, C. S. Kang, J.-H. Woo, X.-Q. Yang, S. M. George, K. H. Oh, S.-J. Cho, and S.-H. Lee, “Unexpected high power performance of atomic layer deposition coated Li[Ni1/3Mn1/3Co1/3]O2 cathodes”, Journal of Power Sources **254**, 190–197 (2014).

[14]Z. Li, R. Xu, S. Deng, X. Su, W. Wu, S. Liu, and M. Wu, “MnS decorated N/S codoped 3D graphene which used as cathode of the lithium-sulfur battery”, Applied Surface Science **433**, 10–15 (2018).

[15]M. Hendrickx, A. Paulus, M. A. Kirsanova, M. K. Van Bael, A. M. Abakumov, A. Hardy, and J. Hadermann, “The Influence of Synthesis Method on the Local Structure and Electrochemical Properties of Li-Rich/Mn-Rich NMC Cathode Materials for Li-Ion Batteries”, Nanomaterials **12**, 2269 (2022).

[16]I. Gomez, D. Mecerreyes, J. A. Blazquez, O. Leonet, H. Ben Youcef, C. Li, J. L. Gómez-Cámer, O. Bondarchuk, and L. Rodriguez-Martinez, “Inverse vulcanization of sulfur with divinylbenzene: Stable and easy processable cathode material for lithium-sulfur batteries”, Journal of Power Sources **329**, 72–78 (2016).

[17]W. Bao, X. Xie, J. Xu, X. Guo, J. Song, W. Wu, D. Su, and G. Wang, “Confined Sulfur in 3 D MXene/Reduced Graphene Oxide Hybrid Nanosheets for Lithium–Sulfur Battery”, Chemistry A European J **23**, 12613–12619 (2017).

[18]H. Zhu, Q. Li, X. Gong, K. Cao, and Z. Chen, “Enhanced High Voltage Performance of Chlorine/Bromine Co-Doped Lithium Nickel Manganese Cobalt Oxide”, Crystals **8**, 425 (2018).

[19]G. Zhou, L. Li, C. Ma, S. Wang, Y. Shi, N. Koratkar, W. Ren, F. Li, and H.-M. Cheng, “A graphene foam electrode with high sulfur loading for flexible and high energy Li-S batteries”, Nano Energy **11**, 356–365 (2015).

[20]K. Xi, P. R. Kidambi, R. Chen, C. Gao, X. Peng, C. Ducati, S. Hofmann, and R. V. Kumar, “Binder free three-dimensional sulphur/few-layer graphene foam cathode with enhanced high-rate capability for rechargeable lithium sulphur batteries”, Nanoscale **6**, 5746–5753 (2014).

[21]X. Ji, K. T. Lee, and L. F. Nazar, “A highly ordered nanostructured carbon–sulphur cathode for lithium–sulphur batteries”, Nature Mater **8**, 500–506 (2009).

[22]Z. Huang, T. Shi, J. Cheng, Y. Liao, H. Ji, Y. Huang, J. Xiang, and L. Yuan, “Balancing sulfur utilization and electrolyte demand in Li-S batteries via porosity-tuned calendaring-driven electrodes”, J. Power Sources **643**, 237052 (2025).

[23]L. Sun, H. Liu, X. Gui, L. Liu, D. Li, X. Sun, Y. Xue, C. Luo, and K. Xu, “A novel sulfur-containing polythiophene cathode active material for reducing shuttle effect in lithium sulfur battery”, Journal of Energy Storage **129**, 117376 (2025).

[24]R. Moschner, M. Gerle, T. Danner, E. K. Simanjuntak, P. Michalowski, A. Latz, M. Nojabaee, A. Kwade, and K. A. Friedrich, “Impact of the Sulfurized Polyacrylonitrile Cathode Microstructure on the Electrochemical Performance of Lithium–Sulfur Batteries”, Advanced Science **12**, 2415436 (2025).

[25]M. Shaibani, M. S. Mirshekarloo, R. Singh, C. D. Easton, M. C. D. Cooray, N. Eshraghi, T. Abendroth, S. Dörfler, H. Althues, S. Kaskel, A. F. Hollenkamp, M. R. Hill, and M. Majumder, “Expansion-tolerant architectures for stable cycling of ultrahigh-loading sulfur cathodes in lithium-sulfur batteries”, Sci. Adv. **6**, eaay2757 (2020).

[26]J. Song, M. L. Gordin, T. Xu, S. Chen, Z. Yu, H. Sohn, J. Lu, Y. Ren, Y. Duan, and D. Wang, “Strong Lithium Polysulfide Chemisorption on Electroactive Sites of Nitrogen-Doped Carbon Composites For High-Performance Lithium–Sulfur Battery Cathodes”, Angew Chem Int Ed **54**, 4325–4329 (2015).

[27]L. Ji, M. Rao, H. Zheng, L. Zhang, Y. Li, W. Duan, J. Guo, E. J. Cairns, and Y. Zhang, “Graphene Oxide as a Sulfur Immobilizer in High Performance Lithium/Sulfur Cells”, J. Am. Chem. Soc. **133**, 18522–18525 (2011).

[28]B. A. Walker, C. O. Plaza-Rivera, S.-S. Sun, W. Lu, J. W. Connell, and Y. Lin, “Dry-pressed lithium nickel cobalt manganese oxide (NCM) cathodes enabled by holey graphene host”, Electrochimica Acta **362**, 137129 (2020).

[29]N. Tran, L. Croguennec, C. Labrugère, C. Jordy, Ph. Biensan, and C. Delmas, “Layered Li[sub 1+x](Ni[sub 0.425]Mn[sub 0.425]Co[sub 0.15])[sub 1-x]O[sub 2] Positive Electrode Materials for Lithium-Ion Batteries”, J. Electrochem. Soc. **153**, A261 (2006).

[30]P. B. Samarasingha, A. Wijayasinghe, M. Behm, L. Dissanayake, and G. Lindbergh, “Development of cathode materials for lithium ion rechargeable batteries based on the system Li(Ni1/3Mn1/3Co(1/3-x)Mx)O2, (M=Mg, Fe, Al and x=0.00 to 0.33)”, Solid State Ionics **268**, 226–230 (2014).

[31]H.-Y. Wang, X.-F. He, S.-L. Mei, Y.-P. Zheng, Y.-W. Feng, N. Li, and Z.-B. Wang, “Boosting the cycling and storage performance of lithium nickel manganese cobalt oxide-based high-rate batteries through cathode manipulation”, Electrochimica Acta **474**, 143566 (2024).

[32]K. Uzun, H. Alolaywi, S. Thapa, B. Frieberg, M. Wang, X. Huang, and Y.-T. Cheng, “Investigating the Effect of Electrode Compositions on Dry-made NMC811 Positive Electrodes”, J. Electrochem. Soc. **171**, 080532 (2024).

[33]H. Dreger, H. Bockholt, W. Haselrieder, and A. Kwade, “Discontinuous and Continuous Processing of Low-Solvent Battery Slurries for Lithium Nickel Cobalt Manganese Oxide Electrodes”, Journal of Elec Materi **44**, 4434–4443 (2015).

[34]S. Abarna, R. Sudha Periathai, R. Pon Vengatesh, and N. Prithivikumaran, “Structural, Electrical, and Electrochemical Characterization of Li1.2Ni0.6-xMgxCo0.3O2 Cathode Materials for Application in Lithium-Ion Batteries”, Journal of Elec Materi **49**, 6622–6630 (2020).

[35]A. Ganesan, A. Varzi, S. Passerini, and M. M. Shaijumon, “Graphene derived carbon confined sulfur cathodes for lithium-sulfur batteries: Electrochemical impedance studies”, Electrochimica Acta **214**, 129–138 (2016).

[36]N. Jayaprakash, J. Shen, S. S. Moganty, A. Corona, and L. A. Archer, “Porous Hollow Carbon@Sulfur Composites for High-Power Lithium–Sulfur Batteries”, Angew Chem Int Ed **50**, 5904–5908 (2011).

[37]L.-J. Liu, Y. Chen, Z.-F. Zhang, X.-L. You, M. D. Walle, Y.-J. Li, and Y.-N. Liu, “Electrochemical reaction of sulfur cathodes with Ni foam current collector in Li-S batteries”, J. Power Sources **325**, 301–305 (2016).

[38]Z. W. Seh, W. Li, J. J. Cha, G. Zheng, Y. Yang, M. T. McDowell, P.-C. Hsu, and Y. Cui, “Sulphur–TiO2 yolk–shell nanoarchitecture with internal void space for long-cycle lithium–sulphur batteries”, Nat Commun **4**, 1331 (2013).

[39]J. Zhang, H. Hu, Z. Li, and X. W. (Lou, “Double-Shelled Nanocages with Cobalt Hydroxide Inner Shell and Layered Double Hydroxides Outer Shell as High-Efficiency Polysulfide Mediator for Lithium–Sulfur Batteries”, Angew Chem Int Ed **55** (2016).

[40]R. Jung, R. Morasch, P. Karayaylali, K. Phillips, F. Maglia, C. Stinner, Y. Shao-Horn, and H. A. Gasteiger, “Effect of Ambient Storage on the Degradation of Ni-Rich Positive Electrode Materials (NMC811) for Li-Ion Batteries”, J. Electrochem. Soc. **165**, A132–A141 (2018).

[41]M. Singh, J. Kaiser, and H. Hahn, “Thick Electrodes for High Energy Lithium Ion Batteries”, J. Electrochem. Soc. **162**, A1196–A1201 (2015).

[42]Z. Lu, D. D. MacNeil, and J. R. Dahn, “Layered Li[Ni[sub x]Co[sub 1-2x]Mn[sub x]]O[sub 2] Cathode Materials for Lithium-Ion Batteries”, Electrochem. Solid-State Lett. **4**, A200 (2001).

[43]P. M. Zehetmaier, F. Zoller, M. Beetz, M. A. Plaß, S. Häringer, B. Böller, M. Döblinger, T. Bein, and D. Fattakhova-Rohlfing, “Nanocellulose-Mediated Transition of Lithium-Rich Pseudo-Quaternary Metal Oxide Nanoparticles into Lithium Nickel Cobalt Manganese Oxide (NCM) Nanostructures”, ChemNanoMat **6**, 618–628 (2020).

[44]S. H. Song, M. Cho, I. Park, J.-G. Yoo, K.-T. Ko, J. Hong, J. Kim, S.-K. Jung, M. Avdeev, S. Ji, S. Lee, J. Bang, and H. Kim, “High-Voltage-Driven Surface Structuring and Electrochemical Stabilization of Ni-Rich Layered Cathode Materials for Li Rechargeable Batteries”, Advanced Energy Materials **10**, 2000521 (2020).

[45]K. Fröhlich, E. Legotin, F. Bärhold, and A. Trifonova, “New large-scale production route for synthesis of lithium nickel manganese cobalt oxide”, J Solid State Electrochem **21**, 3403–3410 (2017).

[46]M. Shiozaki, H. Yamashita, Y. Hirayama, T. Ogami, and K. Kanamura, “Blending Lithium Nickel Manganese Cobalt Oxide with Lithium Iron Manganese Phosphate as Cathode Materials for Lithium-ion Batteries with Enhanced Electrochemical Performance”, Electrochemistry **91**, 077007–077007 (2023).

[47]H. Zheng, Q. Sun, G. Liu, X. Song, and V. S. Battaglia, “Correlation between dissolution behavior and electrochemical cycling performance for LiNi1/3Co1/3Mn1/3O2-based cells”, Journal of Power Sources **207**, 134–140 (2012).
